# Root JA Induction Modifies Glucosinolate Profiles and Increases Subsequent Aboveground Resistance to Herbivore Attack in *Cardamine hirsuta*

**DOI:** 10.3389/fpls.2018.01230

**Published:** 2018-08-21

**Authors:** Moe Bakhtiari, Gaétan Glauser, Sergio Rasmann

**Affiliations:** ^1^Laboratory of Functional Ecology, Institute of Biology, University of Neuchâtel, Neuchâtel, Switzerland; ^2^Neuchâtel Platform of Analytical Chemistry, Neuchâtel, Switzerland

**Keywords:** belowground-aboveground priming, glucosinolates, insect resistance, plant-mediated above-belowground interaction, plant chemical defenses, phytohormones

## Abstract

Alteration and induction of plant secondary metabolites after herbivore attack have been shown in almost all the studied plant species. Induction can be at the local site of damage, or systemic, such as from roots to shoots. In addition to immediate induction, previous herbivore bouts have been shown to “prime” the plants for a stronger and faster response only after a subsequent attack happens. Whereas several studies revealed a link between root herbivory and increased resistance against aboveground (AG) herbivory, the evidence of root defense priming against subsequent AG herbivory is currently lacking. To address this gap, we induced *Cardamine hirsuta* roots by applying jasmonic acid (JA), and, after a time lag, we subjected both control and JA-treated plants to AG herbivory by the generalist herbivore *Spodoptera littoralis*. We addressed the effect of root JA addition on AG herbivore resistance by measuring larval weight gain and tested the effect of root induction on abundance and composition of glucosinolates (GSLs) in shoots, prior, and after subsequent herbivory. We observed a strong positive effect of root induction on the resistance against AG herbivory. The overall abundance and identity of GSLs was globally affected by JA induction and by herbivore feeding, independently, and we found a significant correlation between larval growth and the shoot GSL profiles only after AG herbivory, 11 days after induction in roots. Contrary to expectations of priming, we observed that JA induction in roots altered the GSLs profile in the leaves that was maintained through time. This initial modification was sufficient to maintain a lower caterpillar weight gain, even 11 days post-root induction. Altogether, we show that prior root defense induction increases AG insect resistance by modifying and maintaining variation in GSL profiles during insect feeding.

## Introduction

Resistance to herbivory in plants is mediated by pre-existing, or herbivore-inducible, physical and chemical barriers (Karban and Baldwin, 1997). Specifically, plants can enhance constitutive levels of defenses, or produce them *de novo*, upon herbivore damage (Agrawal et al., 1999). In addition, previous incidents of herbivory do not directly increase defenses but can “prime” plants for a faster and stronger response against subsequent attackers (van Hulten et al., 2006; Ton et al., 2007; Pieterse et al., 2013). Plant defense orchestration is mediated by several plant hormones (Pieterse et al., 2014), of which salicylic acid (SA), jasmonic acid (JA), and ethylene (ET) are the most important, but other phytohormones, such as abscisic acid (ABA), gibberellins, auxins, and cytokinins have more recently been described as important defense regulators as well (van Hulten et al., 2006; Giron et al., 2013). Generally, the plant hormone JA is a key player in the regulation of induced plant responses against chewing herbivores such as beetles and caterpillars (Farmer et al., 2003; Howe and Jander, 2008).

While previous studies of plant-mediated interactions with herbivores have mostly focused on locally infested tissues, it is now known that defense activation can spread systemically through the plant and can even cross the root–shoot divide (Bezemer et al., 2003; Bezemer and van Dam, 2005; Heil and Ton, 2008; Rasmann and Agrawal, 2008). Several studies have demonstrated the crucial role of JA in mediating below- and above-ground (BG and AG thereafter) interactions (Erb et al., 2008; Soler et al., 2013; Fragoso et al., 2014). For instance, exogenous JA exposure to BG or AG parts of a plant can systemically induce defense responses in roots or leaves, respectively (van Dam et al., 2004; van Dam and Oomen, 2008). Therefore, when specifically looking from the root to shoot, root herbivory could negatively affect the performance of leaf-chewing insects by inducing a systemic increase in secondary metabolites (Bezemer et al., 2003; Soler et al., 2005; van Dam et al., 2005; Staley et al., 2007; Erb et al., 2009a,b). BG insect herbivory, or JA application, in some studies, increased defense compound (e.g., glucosinolates (GSLs)) levels in shoots (Griffiths et al., 1994; van Dam et al., 2004; van Dam and Oomen, 2008; Qiu et al., 2009; Pierre et al., 2012, 2013). However, other studies demonstrated that BG induction resulted in a decrease (van Dam et al., 2005), or had no effect on secondary metabolites levels (van Dam and Raaijmakers, 2006; Pierre et al., 2012; Tytgat et al., 2013). This suggests that plant defense induction in the roots could reduce herbivore pressure AG by immediately increasing shoot defenses, (van Dam et al., 2001, van Dam et al., 2004), or by priming the plants for a subsequent stronger response induction only after the shoot herbivore is on the plant.

Stimuli such as previous herbivory, egg deposition, or volatiles from herbivore-infested adjacent plants have been shown to prime JA-mediated anti-herbivore defenses (Rasmann et al., 2012; Vos et al., 2013; Bandoly et al., 2015; Erb et al., 2015). Although, several studies indicate that root herbivory increases the resistance against shoot herbivores (Bezemer et al., 2003; Hol et al., 2004; Soler et al., 2005; van Dam et al., 2005), studies investigating the importance of JA-dependent priming through induction of GSLs in AG-BG context are scarce. For instance, it has been shown that root herbivory by *Delia radicum* primed *Brassica nigra* leaves against subsequent leaf herbivory by *Pieris rapae*, which resulted in stronger increase of AG chemical defenses compared to levels prior to leaf herbivory (van Dam et al., 2005). In contrast, Soler et al. (2005) found no clear effect of BG herbivory on chemical defenses in *B. nigra* leaves attacked by *Pieris brassicae*.

The aim of this study was to explore the JA-dependent root induction effect on subsequent AG herbivore attack. The idea being that root induction by JA would not result in immediate AG changes in secondary metabolites, but that AG priming of defenses – and subsequent increased plant resistance against the herbivore – would only be visible if, after a delay of few days, an herbivore would attack the plant (Martinez-Medina et al., 2016). We tested this hypothesis using a wild Brassicaceae species, the hairy bittercress *Cardamine hirsuta*, and a generalist noctuid butterfly caterpillar *Spodoptera littorali*s. In Brassicaceae plants, GSLs, sulfur- and nitrogen-containing plant secondary metabolites, are the main defensive compounds conferring plant resistance against insect herbivores (Howe and Jander, 2008). Induction by JA or herbivory has been shown to increase the concentration of GSLs in several systems (Papadopoulou and van Dam, 2016) and decrease the performance of generalist herbivores in particular (Bodenhausen and Reymond, 2007).

We specifically had the following questions: (i) does root induction by JA affect plants’ resistance against subsequent shoot herbivory? (ii) does root JA application affect the amount and composition of GSLs in leaves prior and after subsequent shoot herbivory? (iii) is there a relationship between GSLs composition before and after herbivory and resistance to herbivory? We expected that root JA application would increase resistance against subsequent AG herbivore attack. We also expected that, in case of priming, JA application would not modify AG GSL composition, but JA effect would only be visible after AG herbivore application.

## Materials and Methods

### Plant and Insect

To address the effect of root priming on AG plant defense and resistance, we used the hairy bittercress, *C. hirsuta* (Brassicaceae), a common weed growing in a variety of habitats in Europe (Pellissier et al., 2016). Seeds were collected from three different natural populations around Neuchâtel in Switzerland in 2016. Seeds from 26 half-sib families (pop A = 9 fam, pop B = 10 fam, and pop C = 7 fam) were germinated in Petri dishes lined with humid filter paper, and one week after germination, six seedlings per family (total of 156 plants) were transplanted independently into plastic potting pots (13 cm width × 10 cm height) filled with 500 ml of sieved soil (1 cm mesh size) mixed with sand in a 3:1 ratio. The soil/sand mixture was sterilized by autoclave. Plants were immediately transferred to a climate-controlled chamber and kept at 16 h/22°C - 8 h/16°C day-night and 50% relative humidity conditions. Plants received nutrients twice a week for three weeks until the beginning of experiment.

We used *S. littoralis* as generalist herbivore insects (obtained from Syngenta, Stein AG, Switzerland). Newly hatched larvae were reared on corn-based artificial diet until the beginning of the experiment. *S. littoralis* is a generalist herbivore, known to feed on species belonging to more than 40 families of plants (Brown and Dewhurst, 1975) and is widely used for performing plant resistance bioassays. In addition, *S. littoralis* has been shown to activate JA-dependent defenses in *Arabidopsis thaliana*, a close relative of *C. hirsuta* (Bodenhausen and Reymond, 2007).

### Experimental Set-Up

After 3 weeks of growth, plants were randomly assigned to two treatment groups. Half of the plants (three replicates per family, *n* = 78) were randomly assigned to the JA treatment, while the other half to the control treatment (three replicates per family, *n* = 78). JA-treated plants received 20 ml of JA solution in roots by adding the solution in the soil, 0.5 cm below the surface. The JA solution consisted of 2.4 μmol (500 μg) of JA (± - JA, Sigma-Aldrich, St Louis, IL, United States) per plant in 10 ml demineralized water and 0.5% EtOH (pH 4.0). The control group of plants received 20 ml of 0.5% EtOH in acid water (pH 3.7 with HCl) in roots for each plant. These amounts were chosen based on previous studies using other brassicaceous plants (van Dam et al., 2004; van Dam and Oomen, 2008).

Four days after the root treatment, two fully expanded new leaves per plant were collected, immediately frozen and stored at -80°C for further chemical analyses. Right after, two 7-day old *S. littoralis* larvae were added to the leaves of each plant. The combined weight of the insects per plant was measured and recorded. Plants were covered with gauze bags to prevent escape or cross-movement of insects between plants. After one week of herbivory (i.e., 11 days post JA treatment – hereafter “after herbivory”), bags were removed, the insects were retrieved from individual plants, and their weights were measured and recorded. We used the formula ln (final weight–initial weight) to determine the insects’ weight gain and plant resistance (i.e., lower weight gain indicate that plants are more resistant). Two fully expanded herbivore-damaged leaves per plant were collected and immediately placed in -80°C for further chemical analyses. After the herbivore treatment, the plants were allowed to complete their life cycle. In the end of the life cycle, AG plant parts were separated from roots, weighted, oven-dried at 40°C for 48 h and weighted to determine their dry biomass.

### Glucosinolate Extraction and Analysis

Plant leaves, harvested prior and after herbivore treatment, were ground to powder using mortars and pestles in liquid nitrogen, and a 100-mg aliquot was weighted in a 1.5-ml Eppendorf tube for glucosinolate extraction. 1.0 ml Methanol: H2O: formic acid (80:19.5:0.5, v/v) were added to the tubes along with 5 glass-beads and the tubes were shaken in a tissuelyser for 4 min at 30 Hz and centrifuged at 12,800 × *g* for 3 min. The supernatant was then transferred to an appropriate vial for liquid chromatography analysis. Glucosinolate identification and quantification was performed using an Acquity UPLC from Waters (Milford, MA, United States) interfaced to a Synapt G2 QTOF from Waters with electrospray ionization, using the separation and identification method as described in (Glauser et al., 2012). We acknowledge that we did not measure GSLs on a set of control plants that never experienced herbivory at time T2 to infer inducibility of GSLs. The reasoning for doing this was not to measure the specific inducibilities for each compound at time T2, but mainly to correlate what the larvae were experiencing at this time point, versus what the larvae initially experienced at time T1.

### Statistical Analysis

All statistical analyses were carried out with R software (R Development Core Team, 2017). To address the priming effect of root JA addition to AG resistance against *S. littoralis*, as well as the total amount of GSLs, we ran linear mixed effect models with insect weight gain and total amount of GSLs as response variables, JA treatment (two levels) as fixed factor, plant biomass as covariate, and plant families nested within population as random factor using the function *lme* in the package *nlme* in R (Pinheiro et al., 2017).

To address how JA application in root would affect the abundance and composition of GSLs in the shoots, we first ran a full-factorial model including the individual GSLs abundance matrix as response variable and time after induction, JA treatment, and families nested within populations as factors using permutational analysis of variance (PERMANOVA) with the *adonis* function in the package *vegan* in R (Oksanen et al., 2017). To take into account the effect of measuring induction of GSLs on the same plants twice, we included plant IDs as “strata” in the *adonis* function. Finally, we also included plant biomass as covariate to control for potential direct effect of biomass on plant chemistry (Züst et al., 2015), as well as larval weight gain to take into account the effect of larval size, and indirectly, weight gain, on GSL production (Raubenheimer and Simpson, 1992; Horton and Redak, 1993). The Bray–Curtis metric was used to calculate a dissimilarity matrix of all compounds among samples for the PERMANOVA.

Finally, we analyzed the relationship between JA-induced GSLs and larval weight gain using the environmental fitting analysis [*envfit* function in *vegan* (Oksanen et al., 2017)] on the NMDS analysis of the chemical compounds (time = after herbivory). When applied to NMDS, the environmental fitting analysis can estimate the strength of the correlation of maximal correlation between the NMDS configuration and the weight gain variable. This approach can be used to indicate whether larval weight gain is associated with particular GSLs, as represented in the NMDS ordination.

## Results

### Effect of JA Treatment on Resistance Against *S. littoralis*

**FIGURE 1:**
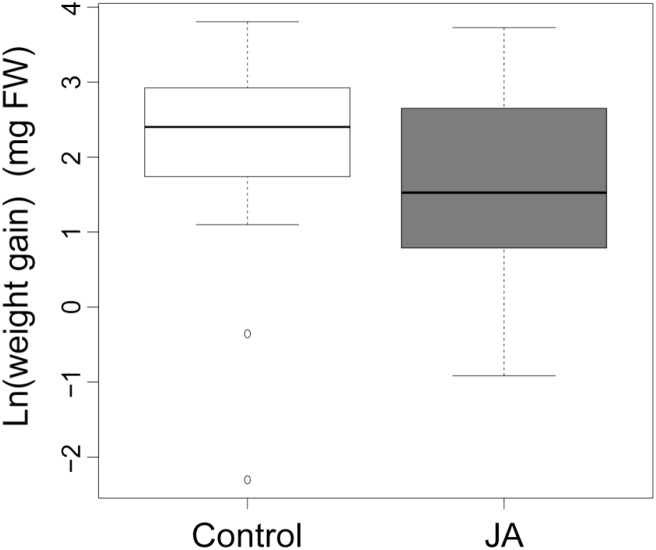
Larval weight gain. The average weight gain of *Spodoptera littoralis* caterpillars feeding on plants that received jasmonic acid (JA) in the roots 4 days prior to the start of herbivory or received no JA in the roots (Control). Weight gain was calculated as the natural logarithm of the difference between final and initial fresh weight. The two boxplots are significantly different (ANOVA, *p* < 0.05).

*Spodoptera littoralis* larvae grew 55% less (absolute weight gain values) on JA-treated plants compared to control plants (**Figure 1**, *F*_1,76_ = 9.67, *p* < 0.003), indicating the significant effect of JA treatment in roots on AG herbivore resistance. We found no effect of plant biomass on larval weight gain (*F*_1,76_ = 0.01, *p* = 0.93).

### Effect of JA Treatment on GSLs

**FIGURE 2:**
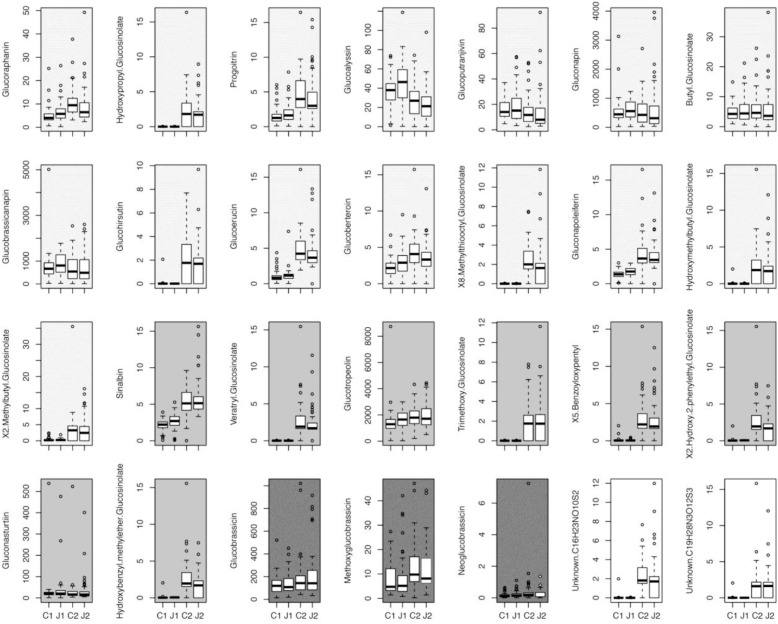
Individual glucosinolate induction. Data show the effect of JA induction in the roots, at two different time points (J1 and J2) and no JA induction (C1 and C2), on individual glucosinolates (ng mg^-1^ FW) levels in the leaves of *Cardamine hirsuta* plants. C2 and J2 also represent 7 days of *Spodoptera littoralis* herbivore attack. Different shadings of gray indicate different classes of GSLs: from light to dark: unknown (white), aliphatic, aromatic, and indole.

**FIGURE 3:**
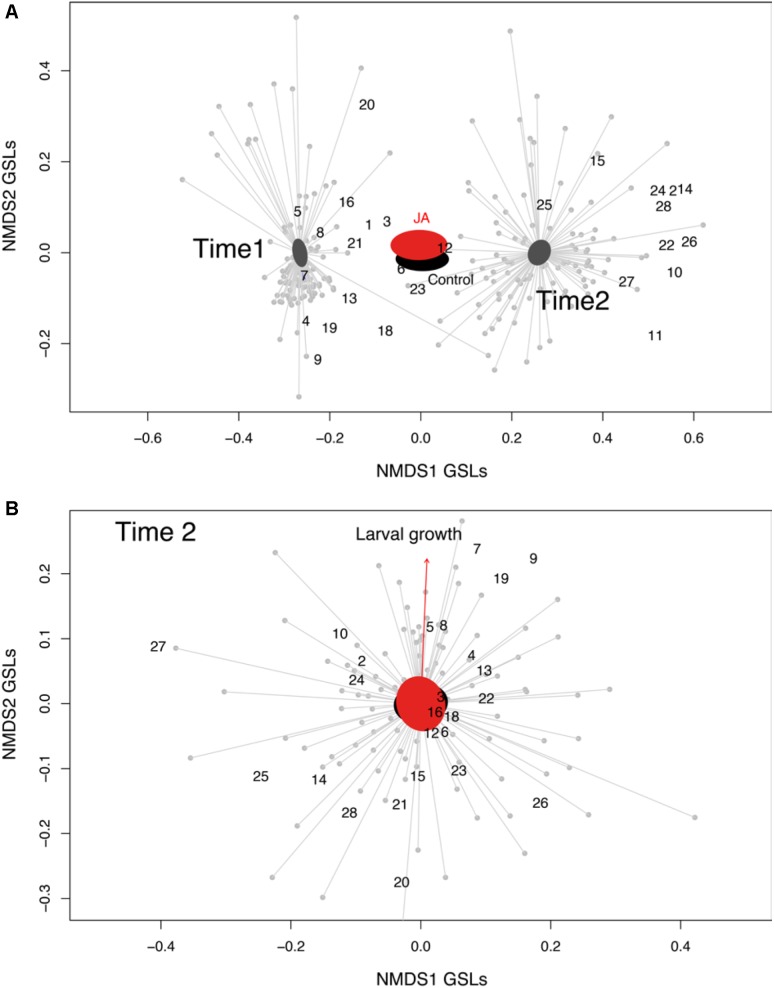
Glucosinolates’ ordination. **(A)** Representation of the non-multidimensional scaling (NMDS) indicating the glucosinolates found in *Cardamine hirsuta* leaves, and their 95% confidence interval ellipses based on the two treatments (root induction with JA, red polygon; and no-induction as the black polygon) at two-time points; Time 1 = 4 days after induction and Time 2 = 11 days after induction (stress value = 0.14, *k* = 2). **(B)** Effect of glucosinolates on larval growth. Representation of the non-multidimensional scaling (NMDS) indicating the glucosinolates found in *C. hirsuta* leaves, and their 95% confidence interval ellipses based on the two treatments (root induction with JA, pink polygon; and no-induction as the green polygon). The projection of the maximal correlation of the larval weight gain vector (from the envfit model) on the NMDS ordination is also shown (red arrow) (stress value = 0.26, *k* = 2). Glucosinolates are: 1 = Glucoraphanin, 2 = Hydroxypropyl-GSL, 3 = Progoitrin, 4 = Glucoalyssin, 5 = Glucoputranjivin, 6 = Sinalbin, 7 = Gluconapin, 8 = Butyl-GSL, 9 = Glucobrassicanapin, 10 = Veratryl-GSL, 11 = Glucohirsutin, 12 = Glucoerucin, 13 = Glucotropeolin, 14 = Trimethoxy-GSL, 15 = 5-Benzoyloxypentyl, 16 = Glucobrassicin, 17 = 2-Hydroxy-2-phenylethyl-GSL, 18 = Glucoberteroin, 19 = Gluconasturtiin, 20 = Methoxyglucobrassicin, 21 = Neoglucobrassicin, 22 = 8-Methylthiooctyl-GSL, 23 = Gluconapoleiferin, 24 = Hydroxymethylbutyl-GSL, 25 = 2-Methylbutyl-GSL, 26 = Unknown-GSL, 27 = Hydroxybenzyl-methylether GSL, and 28 = Unknown-GSL.

The GSLs profile of the *C. hirsuta* leaves, harvested four (before herbivory) and 11 days after root induction (after herbivory), consisted of 28 GSL compounds: 15 aliphatic-GSLs, 8 aromatic-GSLs, 3 indole-GSLs, and 2 unknown-GSLs (**Figure 2** and **Supplementary Table S1**). Total levels of GSLs were only affected by herbivore damage over the 7 days period of feeding, in which, after herbivory, plants produced 10% more GSLs than 4 day post-induction (i.e., measures taken 4 and 11 days after JA treatment) (mixed effect model; Time effect: *F*_1,179_ = 4.81, *p* = 0.02). The PERMANOVA showed that the abundance and diversity of GSLs were globally affected by JA treatment and by one week of continuous damage by AG herbivores (**Figure 3A** and **Table 1**), however, we found no interaction between time and JA induction (**Figure 3A**). We also found that the maternal family background affected the GSLs production, indicating that the genetic background influences the magnitude of GSLs production in shoots after root JA induction and AG herbivory (**Table 1**). Finally, we found that overall; plant biomass was affecting GSLs production in shoots of *C. hirsuta* plants (significant at global GSL levels and significant for 25 of the individual compounds) (**Table 1** and **Supplementary Table S1**). Moreover, specifically, we found interaction between time and induction by JA in five of the individual GSL compounds (3 aliphatic and 2 aromatics), suggesting that despite the pattern observed at the global GSL levels the production of these compounds between JA-treated and control plants depended on time (**Figure 2** and **Supplementary Table S1**).

**Table 1:** Two-way permutation ANOVA table for measuring the effect of JA induction in roots and time after induction on the GSLs matrix of *Cardamine hirsuta* plants.

Factor	*df*	Mean SQ	*F* value	*R*^2^	*p*
Time	1	0.57	7.29	0.03	0.001^∗∗∗^
JA (induction treatment)	1	0.1	1.24	0.005	0.002^∗∗^
Time × JA	1	0.15	1.85	0.008	0.180
Families	10	0.15	1.84	0.08	0.001^∗∗∗^
Families/population	15	0.15	1.89	0.12	0.004^∗∗^
Larval weight gain	1	0.07	0.83	0.003	0.001^∗∗∗^
Plant biomass	1	0.38	4.87	0.02	0.002^∗∗^
Residuals	175	0.08		0.74	


*Significance codes: ^∗∗∗^*p* = 0.001, ^∗∗^*p* = 0.01, ^∗^*p* = 0.05.*

### Effect of GSLs Matrix and Time on Larval Growth

After correlating the larval growth with the GSLs ordination matrix (NMDS), we found that GSLs profiles of the shoots significantly correlated with larval growth only after herbivory (**Figure 3B**, envfit analysis, *r^2^* = 0.07, *p* = 0.02), while such a correlation was not present in time 4 days (*r^2^* = 0.01, *p* = 0.44).

## Discussion

**FIGURE 4:**
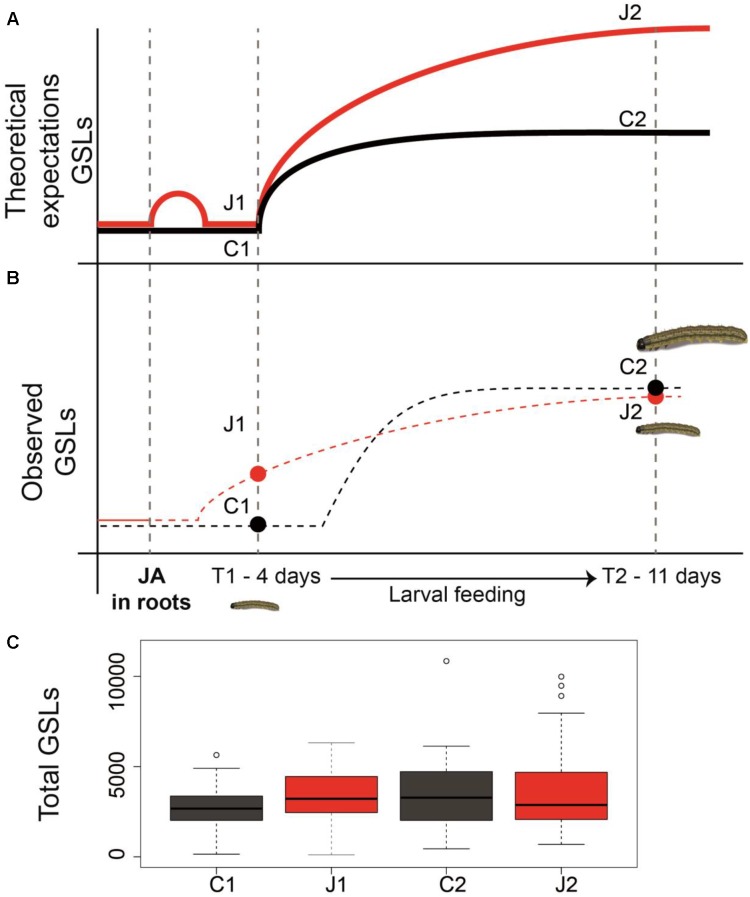
Priming of defenses and resistance in plants. Panel **(A)** is showing the theoretical expectations of defense priming in plants based on the literature. Panel **(B)** shows the conceptual model of priming that was observed in this paper as well as the resistance bioassay with *Spodoptera littoralis* caterpillars. Dots represent the observed values of total GSL sampled at two time points. The dotted lines represent hypothetical GSL induction dynamics. Panel **(C)** shows how total glucosinolates levels (ng mg^-1^ FW) vary across time and based on the two treatments of JA induction in the roots (J1 and J2), and no JA induction (C1 and C2).

Alteration and induction of plant secondary metabolites in response to herbivore attack have been shown in almost all the studied plant species. However, whereas several studies demonstrate that root herbivory results in increased resistance against AG herbivory (Bezemer et al., 2003; Hol et al., 2004; Soler et al., 2005; van Dam et al., 2005), the importance of root defense priming against subsequent AG herbivory has not been thoroughly investigated in this context. In this study, we expected a priming effect of JA application in the roots (**Figure 4A**); however, we observed that JA in roots induced an initial modification in the GSLs identity and quantity in the leaves that was maintained through time. This initial modification was sufficient to increase plants’ resistance against AG herbivory, even 11 days post-root induction (**Figure 4B**). Altogether, these results indicate that root defense induction increases AG resistance to herbivory in *C. hirsuta*, by immediately modifying the GSL profiles in the leaves.

### Effect of JA Treatment on Resistance Against *S. littoralis*

Jasmonic acid application in roots reduced *S. littoralis* weight gain. Overall, our results follows the general trend reported in the literature predicting that hormonal induction of BG tissues increases AG resistance against shoot herbivores (Erb et al., 2011; Papadopoulou and van Dam, 2016) and complement several other studies indicating that root herbivory results in increased resistance against AG herbivory (Bezemer et al., 2003; Hol et al., 2004; Soler et al., 2005; van Dam et al., 2005). For example, it has previously been shown that JA treatment of roots in *Brassica oleracea* negatively affected the growth and survival of a generalist *Mamestra brassicae* (van Dam and Oomen, 2008). This trend is however not universal. For example, JA treatment of roots have shown to be ineffective against *M. brassicae* in field-grown cultivated *B. oleracea* plants (Pierre et al., 2013), which could be explained by the differences between flowers’ (the broccolis) and leaves’ chemistry, and induction therein.

Although, in this study, we used JA to mimic the effect of BG herbivory, it has been clearly shown that JA-induced responses follow similar pattern of induction by BG herbivory. Indeed, the effect of BG herbivory on generating induced response in shoots has been amply demonstrated (van Dam et al., 2005; van Dam and Raaijmakers, 2006; Pierre et al., 2012, 2013), and several studies have shown the same induction pattern in roots caused also by application of JA in roots (van Dam et al., 2004; van Dam and Oomen, 2008; Pierre et al., 2012, 2013). Although, in one study, root infestation with *D. radicum* maggots resulted in weaker systemic responses than JA application (Pierre et al., 2013). Nevertheless, it should be noted that alterations in other plant chemicals, such as induced non-GSLs secondary metabolites, as well as reallocation of primary metabolites between root and shoots may contribute to the observed herbivore responses to induced plants (Jansen et al., 2008; van Dam and Oomen, 2008; Poelman et al., 2010; Pierre et al., 2012). Interestingly, we also found that plant biomass *per se* did not influence the insect weight gain, indicating the larval weight gain was independent of plant size, thus likely mainly mediated by plant defensive traits.

### Effect of JA Treatment on GSLs

We found that the GSLs profiles were different between control and JA-treated plants before and after a week of herbivory. While ontogeny could play a strong role in affecting GSLs production (Barton and Koricheva, 2010), we observed that the total GSLs differences between treatments were maintained through the 7-day time difference. In contrast to that, we found high specificity in how the individual compounds responded to JA root induction and herbivory. Specifically, the production of five individual GSLs: glucoraphanin, glucoalyssin, glucoberteroin, 2-hydroxy-2-phenylethyl GSL, and hydroxybenzyl-methylether GSL across different treatments were significantly affected during herbivore feeding (i.e., significant JA × Time interaction in **Supplementary Table S1**). This suggests that JA induction had significant different effects on the amount of these compounds before and after AG herbivory. For the latter two compounds, we observed the both effect of time and induction as well as interaction between time and JA induction. These results suggest that changes in the complex combinatorial GSL matrix are driving variation in insect resistance, rather than the simple measure of total GSLs contents (**Figure 4C**). Our results are in line with the literature showing that while BG herbivory, or root induction by JA, results in increase in total levels of GSLs in shoots (Griffiths et al., 1994; van Dam et al., 2004; Soler et al., 2005; van Dam and Raaijmakers, 2006; van Dam and Oomen, 2008; Jansen et al., 2009; Qiu et al., 2009; Pierre et al., 2012), others have observed no changes in total GSLs when plants (broccoli) where induced in roots either by JA or *D. radicum* (Pierre et al., 2013). Therefore, both the total amount and the individual-level variation of GSLs could affect resistance against herbivores.

We found a significant effect of plant biomass on GSLs production in plant leaves, a common phenomenon when studying secondary metabolite production in plants (Traw, 2002; Glynn et al., 2003; Züst et al., 2015). We also found a significant effect of larval biomass on the glucosinolate matrix (**Table 1**), suggesting that the potential variation in insect weight gain (i.e., insects that grew more were also eating more) between treatments could potentially also drive the observed variation in the GSL matrix. Furthermore, the observed strong family level variation in induction of GSLs in shoots, after root induction and AG herbivory, is particularly interesting. Such results suggest a great potential for selection on BG-AG induction *per se*, which in turn set the stage for evolution of plant-mediated BG-AG interactions.

### Effect of GSLs Matrix and Time on Larval Growth; Is It Priming?

The larval growth was affected by GSLs profile of the shoots only after herbivory, while such a correlation was not present in 4-day time. These results, while only correlative, point toward the possibility of priming for defense in BG-AG context which indicates that induction in one compartment should increase the resistance to subsequent herbivory in distant tissues (Erb et al., 2008). However, we take the evidence for potential priming with caution.

Despite the emerging evidence on the effect of root herbivory on enhanced resistance against AG herbivory, the importance of priming in BG-AG concept has generally investigated on local tissues. For example, priming by green leaf volatiles against leaf herbivory in maize plants (Engelberth et al., 2004; Ton et al., 2007), priming of feeding-induced defense triggered by ovipositioning against subsequent larval feeding (Bandoly et al., 2015, 2016), and priming of anti-herbivore defense by exposure of plants to volatiles released from feeding-damaged neighboring plants (Engelberth et al., 2004; Heil and Kost, 2006; Heil and Silva Bueno, 2007; Frost et al., 2008). Within the BG-AG framework, we have no clear evidence of priming, so far. Perhaps, the best example to date has shown that *D. radicum* attack of the roots resulted in lower initial GSL levels in the shoot of *B. nigra*, followed by a strong increase in leaf glucosinolate levels upon AG herbivory by *P. rapae*, suggesting that *B. nigra* leaves were primed for defense after root induction (van Dam et al., 2005).

As proposed by Martinez-Medina et al. (2016), in order to assess the presence of defense priming in plants, defense-primed plants should possess certain characteristic key features:(i) memory, (ii) more robust defense, and (iii) low fitness cost and better performance. In our study, in order to reveal whether the information of priming stimulus (JA induction) was stored in plants, we applied two sequential incidents: a priming event followed by the AG herbivore challenge. In response to stressor, JA-treated plants (primed) exhibited higher resistance in a more robust manner compared to control plants (unprimed). As outlined in **Figure 4A**, the theoretical expectation of priming by induction suggests a slight and transient induction of defense traits, by priming stimulus, during the time between the perception of the priming stimulus and the triggering stress. This moderate induction should return to nearly basal levels prior to the triggering stress (see **Figure 4A**; Martinez-Medina et al., 2016). In line with this idea, we found a non-significant induction of total GSLs levels between JA-treated plants versus control plants at time T1. During the larval feeding, theoretically, primed plants should exhibit stronger defense response (**Figure 4A**; higher GSLs in this model); however, our results show no changes in GSLs between treated and non-treated plants (**Figures 4B,C**). This might be due to the fact that the allocation of defenses from root to shoots happened rather quickly upon induction in roots and root-induced plants invested their optimal defense energy quickly upon induction. Given such a scenario was in play; we could expect to observe such a decline at time T2. Perhaps if GSLs measurements were taken at rather earlier stage after AG herbivory, our results would deviate less from the theory expectations. Because priming often involves a faster reaction upon attack, it is crucial to take measurements at multiple time points to detect its occurrence (Engelberth et al., 2004; Ton et al., 2007). Nevertheless, decline of larval weight on JA-induced plants and the correlation between larval weight gain and GSL levels only at time T2 may suggest that the variation of GSL levels between the treatments were more pronounced prior to our measurement at time T2. Therefore, we suggest that the modification of the GSLs profiles upon subsequent AG herbivory and during larval feedings could explain the *S. littoralis* lower weight gain on induced plants. Interestingly, individual GSL induction was overall rather small (see **Supplementary Table S1**) compared to studies showing a clear link between GSL induction and resistance (see,e.g., knock-out mutant studies using *A. thaliana*) (Schlaeppi et al., 2008; Schweizer et al., 2013, 2017). However, other studies have shown weak-to-none GSL induction, while leading to strong induced resistance (Rasmann et al., 2012). Therefore, induction patterns of GSL are indeed informative but they can only give a partial picture of all the potential metabolic changes that happen during the priming phase, which eventually affect insect resistance.

Furthermore, although measuring the fitness cost of priming was outside of the intention of our study, we can argue that JA-treated (primed) plants performed better than control plants on a basis that larvae grew less, and potentially consumed less plant biomass. Our design could only partially address all the criteria for detecting the presence of priming, but the obtained results point toward this direction (Martinez-Medina et al., 2016). In order to evaluate the certainty of priming, further studies should take into consideration the fitness costs, plant lifetime performance, as well as molecular analysis to detect the primed state using molecular markers, such as measuring the expression of defense marker genes and hormone levels (Engelberth et al., 2004; Ton et al., 2007). Therefore, to step beyond the growing literature on plant-mediated BG-AG interactions that vary in space and time, we need to further develop novel model system that can be transposed in field situations.

## Author Contributions

MB performed the experiments, analyzed the data, and wrote the manuscript. SR supervised the experiment, analyzed the data, and wrote the manuscript. GG assisted with chemical analysis.

## Conflict of Interest Statement

The authors declare that the research was conducted in the absence of any commercial or financial relationships that could be construed as a potential conflict of interest.The reviewer CR and handling Editor declared their shared affiliation.
