# Supplementary material for: Root JA Induction Modifies Glucosinolate Profiles and Increases Subsequent Aboveground Resistance to Herbivore Attack in Cardamine hirsuta
Source: Front Plant Sci. 2018 Aug 21;9:1230. doi: 10.3389/fpls.2018.01230 (PMC6110943; doi:10.3389/fpls.2018.01230)
Supplement: Supplementary file 1 [file Table_1.DOCX]

**Table S1.** Two-way ANOVA table of individual glucosinolates (GSLs) for measuring the effect of root JA induction and time after induction. The model was added with plant biomass as covariate and maternal families of plants nested within populations as random factors.

| **Compounds** | **Factors** | **Df** | **F value** | **P-value** | ^a^ |
| --- | --- | --- | --- | --- | --- |
| **Glucoraphanin** | Time | 1 | 39.28 | <0.001 | *** |
|  | JA | 1 | 0.05 | 0.819 |  |
|  | Biomass | 1 | 36.73 | <0.001 | *** |
|  | Fam | 10 | 2.41 | 0.01 | ** |
|  | Time*JA | 1 | 5.08 | 0.025 | ** |
|  | Fam:Pop | 15 | 4.36 | <0.001 | *** |
|  | Residuals | 198 |  |  |  |
| **Hydroxypropyl glucosinolate** | Time | 1 | 96.36 | <0.001 | *** |
|  | JA | 1 | 0.93 | 0.337 |  |
|  | Biomass | 1 | 13.27 | <0.001 | *** |
|  | Fam | 10 | 0.94 | 0.495 |  |
|  | Time*JA | 1 | 0.73 | 0.393 |  |
|  | Fam:Pop | 15 | 1.29 | 0.209 |  |
|  | Residuals | 198 |  |  |  |
| **Progoitrin** | Time | 1 | 107.75 | <0.001 | *** |
|  | JA | 1 | 0.08 | 0.779 |  |
|  | Biomass | 1 | 46.51 | <0.001 | *** |
|  | Fam | 10 | 2.47 | 0.008 | ** |
|  | Time*JA | 1 | 1.24 | 0.266 |  |
|  | Fam:Pop | 15 | 3.71 | <0.001 | *** |
|  | Residuals | 198 |  |  |  |
| **Glucoalyssin** | Time | 1 | 42.86 | <0.001 | *** |
|  | JA | 1 | 1.13 | 0.29 |  |
|  | Biomass | 1 | 2.03 | 0.156 |  |
|  | Fam | 10 | 2.9 | 0.002 | ** |
|  | Time*JA | 1 | 7.85 | 0.006 | ** |
|  | Fam:Pop | 15 | 5.57 | <0.001 | *** |
|  | Residuals | 198 |  |  |  |
| **Glucoputranjivin** | Time | 1 | 3.07 | 0.081 | ° |
|  | JA | 1 | 0.82 | 0.368 |  |
|  | Biomass | 1 | 7.63 | 0.006 | ** |
|  | Fam | 10 | 3.49 | <0.001 | *** |
|  | Time*JA | 1 | 0.93 | 0.335 |  |
|  | Fam:Pop | 15 | 1.96 | 0.02 | ** |
|  | Residuals | 198 |  |  |  |
| **Gluconapin** | Time | 1 | 0.08 | 0.775 |  |
|  | JA | 1 | 0.97 | 0.325 |  |
|  | Biomass | 1 | 9.76 | 0.002 | ** |
|  | Fam | 10 | 1.11 | 0.353 |  |
|  | Time*JA | 1 | 0.06 | 0.814 |  |
|  | Fam:Pop | 15 | 1.63 | 0.069 | ° |
|  | Residuals | 198 |  |  |  |
| **Butyl glucosinolate** | Time | 1 | 1.95 | 0.164 |  |
|  | JA | 1 | 0.14 | 0.71 |  |
|  | Biomass | 1 | 15.38 | <0.001 | *** |
|  | Fam | 10 | 3.83 | <0.001 | *** |
|  | Time*JA | 1 | 0.61 | 0.435 |  |
|  | Fam:Pop | 15 | 1.77 | 0.041 | ** |
|  | Residuals | 198 |  |  |  |
| **Glucobrassicanapin** | Time | 1 | 0.39 | 0.531 |  |
|  | JA | 1 | 0.91 | 0.341 |  |
|  | Biomass | 1 | 0.43 | 0.511 |  |
|  | Fam | 10 | 1.79 | 0.065 | ° |
|  | Time*JA | 1 | 0.59 | 0.444 |  |
|  | Fam:Pop | 15 | 2.15 | 0.009 | ** |
|  | Residuals | 198 |  |  |  |
| **Glucohirsutin** | Time | 1 | 116.11 | <0.001 | *** |
|  | JA | 1 | 1.06 | 0.305 |  |
|  | Biomass | 1 | 3.3 | 0.071 | ° |
|  | Fam | 10 | 0.41 | 0.941 |  |
|  | Time*JA | 1 | 0.51 | 0.476 |  |
|  | Fam:Pop | 15 | 1.77 | 0.04 | ** |
|  | Residuals | 198 |  |  |  |
| **Glucoerucin** | Time | 1 | 240 | <0.001 | *** |
|  | JA | 1 | 0.98 | 0.323 |  |
|  | Biomass | 1 | 26.93 | <0.001 | *** |
|  | Fam | 10 | 1.51 | 0.139 |  |
|  | Time*JA | 1 | 2.2 | 0.14 |  |
|  | Fam:Pop | 15 | 1.54 | 0.095 | ° |
|  | Residuals | 198 |  |  |  |
| **Glucoberteroin** | Time | 1 | 38.01 | <0.001 | *** |
|  | JA | 1 | 0.01 | 0.942 |  |
|  | Biomass | 1 | 3.12 | 0.079 | ° |
|  | Fam | 10 | 1.1 | 0.363 |  |
|  | Time*JA | 1 | 8.34 | 0.004 | ** |
|  | Fam:Pop | 15 | 1.6 | 0.076 | ° |
|  | Residuals | 198 |  |  |  |
| **8-Methylthiooctyl glucosinolate** | Time | 1 | 121.34 | <0.001 | *** |
|  | JA | 1 | 1.79 | 0.182 |  |
|  | Biomass | 1 | 14.51 | <0.001 | *** |
|  | Fam | 10 | 1.65 | 0.096 | ° |
|  | Time*JA | 1 | 1.35 | 0.247 |  |
|  | Fam:Pop | 15 | 1.75 | 0.044 | ** |
|  | Residuals | 198 |  |  |  |
| **Gluconapoleiferin** | Time | 1 | 151.36 | <0.001 | *** |
|  | JA | 1 | 0.02 | 0.882 |  |
|  | Biomass | 1 | 12.99 | <0.001 | *** |
|  | Fam | 10 | 0.88 | 0.552 |  |
|  | Time*JA | 1 | 2.26 | 0.135 |  |
|  | Fam:Pop | 15 | 1.32 | 0.19 |  |
|  | Residuals | 198 |  |  |  |
| **Hydroxymethylbutyl glucosinolate** | Time | 1 | 100.17 | <0.001 | *** |
|  | JA | 1 | 0.22 | 0.637 |  |
|  | Biomass | 1 | 22.24 | <0.001 | *** |
|  | Fam | 10 | 2.07 | 0.028 | ** |
|  | Time*JA | 1 | 0.01 | 0.918 |  |
|  | Fam:Pop | 15 | 0.88 | 0.591 |  |
|  | Residuals | 198 |  |  |  |
| **2-Methylbutyl glucosinolate** | Time | 1 | 62.05 | <0.001 | *** |
|  | JA | 1 | 0.18 | 0.672 |  |
|  | Biomass | 1 | 13.08 | <0.001 | *** |
|  | Fam | 10 | 1 | 0.445 |  |
|  | Time*JA | 1 | 0.06 | 0.81 |  |
|  | Fam:Pop | 15 | 0.95 | 0.51 |  |
|  | Residuals | 198 |  |  |  |
| **Sinalbin** | Time | 1 | 232.87 | <0.001 | *** |
|  | JA | 1 | 4.71 | 0.031 | ** |
|  | Biomass | 1 | 17.68 | <0.001 | *** |
|  | Fam | 10 | 1.34 | 0.212 |  |
|  | Time*JA | 1 | 0.07 | 0.785 |  |
|  | Fam:Pop | 15 | 2 | 0.017 | ** |
|  | Residuals | 198 |  |  |  |
| **Veratryl glucosinolate** | Time | 1 | 123.06 | <0.001 | *** |
|  | JA | 1 | 1.17 | 0.28 |  |
|  | Biomass | 1 | 17.01 | <0.001 | *** |
|  | Fam | 10 | 1 | 0.448 |  |
|  | Time*JA | 1 | 0.87 | 0.351 |  |
|  | Fam:Pop | 15 | 1.41 | 0.144 |  |
|  | Residuals | 198 |  |  |  |
| **Glucotropeolin** | Time | 1 | 10.33 | 0.002 | ** |
|  | JA | 1 | 1.5 | 0.222 |  |
|  | Biomass | 1 | 0.34 | 0.563 |  |
|  | Fam | 10 | 1.64 | 0.098 | ° |
|  | Time*JA | 1 | 0.11 | 0.745 |  |
|  | Fam:Pop | 15 | 1.62 | 0.071 | ° |
|  | Residuals | 198 |  |  |  |
| **Trimethoxy glucosinolate** | Time | 1 | 106.82 | <0.001 | *** |
|  | JA | 1 | 0.51 | 0.477 |  |
|  | Biomass | 1 | 11.52 | <0.001 | *** |
|  | Fam | 10 | 0.67 | 0.751 |  |
|  | Time*JA | 1 | 0.74 | 0.391 |  |
|  | Fam:Pop | 15 | 0.55 | 0.906 |  |
|  | Residuals | 198 |  |  |  |
| **5-Benzoyloxypentyl** | Time | 1 | 144.03 | <0.001 | *** |
|  | JA | 1 | 0.21 | 0.649 |  |
|  | Biomass | 1 | 31.15 | <0.001 | *** |
|  | Fam | 10 | 1.05 | 0.407 |  |
|  | Time*JA | 1 | 0.01 | 0.93 |  |
|  | Fam:Pop | 15 | 1.23 | 0.253 |  |
|  | Residuals | 198 |  |  |  |
| **2-Hydroxy-2-phenylethyl glucosinolate** | Time | 1 | 103.77 | <0.001 | *** |
|  | JA | 1 | 3.89 | 0.05 | ° |
|  | Biomass | 1 | 13.92 | <0.001 | *** |
|  | Fam | 10 | 0.83 | 0.603 |  |
|  | Time*JA | 1 | 3.02 | 0.084 | ° |
|  | Fam:Pop | 15 | 0.98 | 0.478 |  |
|  | Residuals | 198 |  |  |  |
| **Gluconasturtiin** | Time | 1 | 0.06 | 0.808 |  |
|  | JA | 1 | 2.69 | 0.103 |  |
|  | Biomass | 1 | 14.11 | <0.001 | *** |
|  | Fam | 10 | 33.82 | <0.001 | *** |
|  | Time*JA | 1 | 0.39 | 0.531 |  |
|  | Fam:Pop | 15 | 65.19 | <0.001 | *** |
|  | Residuals | 198 |  |  |  |
| **Hydroxybenzylmethylether glucosinolate** | Time | 1 | 103.77 | <0.001 | *** |
|  | JA | 1 | 3.89 | 0.05 | ° |
|  | Biomass | 1 | 13.92 | <0.001 | *** |
|  | Fam | 10 | 0.83 | 0.603 |  |
|  | Time*JA | 1 | 3.02 | 0.084 | ° |
|  | Fam:Pop | 15 | 0.98 | 0.478 |  |
|  | Residuals | 198 |  |  |  |
| **Glucobrassicin** | Time | 1 | 11.84 | <0.001 | *** |
|  | JA | 1 | 0.64 | 0.423 |  |
|  | Biomass | 1 | 42.25 | <0.001 | *** |
|  | Fam | 10 | 1.18 | 0.308 |  |
|  | Time*JA | 1 | 0.08 | 0.779 |  |
|  | Fam:Pop | 15 | 0.83 | 0.638 |  |
|  | Residuals | 198 |  |  |  |
| **Methoxyglucobrassicin** | Time | 1 | 16.67 | <0.001 | *** |
|  | JA | 1 | 0.08 | 0.772 |  |
|  | Biomass | 1 | 50.99 | <0.001 | *** |
|  | Fam | 10 | 1.82 | 0.059 | ° |
|  | Time*JA | 1 | 0.02 | 0.895 |  |
|  | Fam:Pop | 15 | 3.21 | <0.001 | *** |
|  | Residuals | 198 |  |  |  |
| **Neoglucobrassicin** | Time | 1 | 4.23 | 0.041 | ** |
|  | JA | 1 | 0.51 | 0.475 |  |
|  | Biomass | 1 | 12.55 | <0.001 | *** |
|  | Fam | 10 | 0.87 | 0.567 |  |
|  | Time*JA | 1 | 2.09 | 0.15 |  |
|  | Fam:Pop | 15 | 0.94 | 0.518 |  |
|  | Residuals | 198 |  |  |  |
| **Unknown 1.C16H23NO10S2** | Time | 1 | 127.98 | <0.001 | *** |
|  | JA | 1 | 0.02 | 0.9 |  |
|  | Biomass | 1 | 12.48 | <0.001 | *** |
|  | Fam | 10 | 1.76 | 0.071 | ° |
|  | Time*JA | 1 | 0.06 | 0.807 |  |
|  | Fam:Pop | 15 | 0.84 | 0.627 |  |
|  | Residuals | 198 |  |  |  |
| **Unknown 2.C19H28N3O12S3** | Time | 1 | 58.45 | <0.001 | *** |
|  | JA | 1 | 0 | 0.997 |  |
|  | Biomass | 1 | 11.19 | <0.001 | *** |
|  | Fam | 10 | 0.89 | 0.547 |  |
|  | Time*JA | 1 | 0.07 | 0.794 |  |
|  | Fam:Pop | 15 | 1.23 | 0.249 |  |
|  | **Residuals** | **198** |  |  |  |

^a^Significance codes: "***" =p < 0.001, "**" = p <0.05, "°" = p < 0.1
